# Detection of Acute Tropical Fever Pathogens via Multiplex Polymerase Chain Reaction: A Cross-Sectional Study to Guide Appropriate Antimicrobial Use in Communities

**DOI:** 10.7759/cureus.95654

**Published:** 2025-10-29

**Authors:** Riffat Ahmed, Seema Irfan, Ammarah Baig, Najia Ghanchi, Farah N Qamar, Afia Zafar

**Affiliations:** 1 Department of Pathology and Laboratory Medicine, Section of Microbiology, Aga Khan University Hospital, Karachi, PAK; 2 Department of Pediatrics, Aga Khan University Hospital, Karachi, PAK

**Keywords:** antimicrobial stewardship, dengue, malaria, multiplex pcr, tropical fever, typhoid

## Abstract

Background: Pakistan faces a substantial burden of tropical diseases, including malaria, dengue, and typhoid fever, particularly in Sindh Province, where recent outbreaks and floods have further increased disease incidence, highlighting the urgent need for improved diagnostics and disease control. Currently, diagnosis relies on multiple laboratory tests, contributing to frequent empirical antimicrobial use due to many undiagnosed or misdiagnosed cases. This study aimed to validate a rapid molecular test for the early diagnosis of malaria, dengue, and typhoid infections, thereby facilitating targeted therapy and reducing empirical antimicrobial use.

Methods: Patients aged three months or older who presented to Aga Khan Hospital clinical laboratories with fever lasting two to seven days were recruited. From a total of 1,123 collected blood samples, 400 were randomly selected for multiplex real-time polymerase chain reaction (PCR) testing. Multiplex PCR was performed alongside standard blood tests for malaria, dengue, and typhoid. Sensitivity, specificity, positive predictive value, and negative predictive value of PCR were calculated and compared with routine tests. Data analysis was conducted using STATA 17.0 (StataCorp, College Station, Texas).

Results: Among the 1,123 samples collected over one year, 400 blood samples were randomly selected for molecular testing. Of these, 211 were PCR positive. The sensitivity for detecting malaria was 92.4%, specificity was 98.4%, and the positive and negative predictive values were 90.7% and 98.6%, respectively. The specificity of PCR for dengue and typhoid was 94.6% and 93%, respectively; however, the sensitivity for both dengue and typhoid was low (35.3% and 25%, respectively).

Conclusion: While multiplex PCR demonstrates high accuracy in detecting malaria, its limited sensitivity for dengue and typhoid highlights the need for more advanced molecular assays. Developing rapid and reliable diagnostic tests is essential to enable targeted treatment and promote antimicrobial stewardship within the community.

## Introduction

Fever of unknown origin without localized signs remains the predominant clinical presentation of endemic tropical infections across subtropical and tropical regions [1]. This clinical syndrome is attributable to a broad spectrum of etiological agents, including enteric fever pathogens such as *Salmonella enterica *serovars Typhi and Paratyphi*,* *Plasmodium spp., Rickettsia spp., Leptospira spp*., as well as arboviruses like chikungunya virus, dengue virus, and West Nile virus, among others. The nonspecific and overlapping symptomatology challenges clinical discrimination, frequently impeding timely and accurate diagnosis.

Pakistan has experienced recurrent and escalating outbreaks of these tropical infections, with dengue virus circulation documented since 1994 and the emergence and spread of extensively drug-resistant (XDR) *Salmonella *Typhi strains since 2019 posing critical public health concerns [2-4]. The persistence and amplification of these outbreaks are spurred by environmental and infrastructural inadequacies, notably suboptimal vector control, contaminated water sources, and insufficient sanitation [5]. Despite progress at the global level, with an estimated 60% decline in malaria mortality from 2000 to 2015, malaria continues to impose disproportionate morbidity and mortality in Pakistan [6]. Specifically, Sindh Province and Karachi city have borne a substantial disease burden, reporting approximately 1.35 million confirmed malaria cases in 2023. Concurrently, dengue cases numbered 6,888, and there were over 5,300 laboratory-confirmed typhoid cases primarily affecting pediatric populations under 15 years of age [7-9]. 

The sequelae of recent severe flooding events have further exacerbated disease transmission dynamics, underscoring an exigent need for enhanced diagnostic capabilities and comprehensive control measures [10]. Resource limitations constraining widespread access to diagnostic modalities in Pakistan frequently culminate in diagnostic delays and reliance on empirical antimicrobial administration. This practice risks suboptimal clinical outcomes and fosters antimicrobial resistance. Accurate, rapid diagnostics are indispensable for guiding targeted therapies, mitigating disease progression, and facilitating antimicrobial stewardship. Furthermore, robust diagnostic data underpin effective epidemiological surveillance critical for evaluating and optimizing national and international health interventions.

Conventional gold standards bear challenges; for example, malaria diagnosis via microscopy is highly operator-dependent and prone to inconsistency, whereas advanced molecular approaches such as nested polymerase chain reaction (PCR) offer improved sensitivity and specificity. The advent of multiplex syndromic testing panels represents a paradigm shift, enabling simultaneous, rapid detection of multiple pathogens accelerating clinical decision-making and improving patient outcomes [11-13]. This study aimed to validate a multiplex PCR panel for the rapid diagnosis of dengue,* Salmonella *Typhoid/Paratyphoid, and malaria in Pakistan, with the goal of supporting targeted treatment and antimicrobial stewardship. Additionally, we assessed demographic and clinical characteristics.

## Materials and methods

Study design and setting

A prospective cross-sectional study was conducted between November 29, 2021, and November 15, 2022, within the clinical microbiology laboratory of Aga Khan Hospital, a tertiary care center located in Karachi, Pakistan.

Participant recruitment and ethical considerations

Patients presenting to the hospital and referred by their attending physicians for standard diagnostic testing for suspected dengue, typhoid/paratyphoid, or malaria were consecutively recruited. Eligibility criteria included individuals aged three months or older, encompassing both pediatric and adult populations presenting with clinical suspicion consistent with enteric fever, dengue, or malaria, but without an alternative confirmed infectious focus. Duplicate or repeat samples and specimens containing heparin were excluded to avoid interference with molecular assays. Verbal informed consent was obtained telephonically in the presence of a witness before data collection, in accordance with institutional ethical standards. Demographic and relevant clinical data were systematically recorded in a secure electronic database. This study was approved by the Ethics Review Committee of Aga Khan University (2021-5997-19722). All participants provided verbal informed consent before enrollment in the study. This research was conducted in accordance with the World Medical Association Declaration of Helsinki.

Sample size determination

Sample size estimation considered an expected disease prevalence of 19% ± 5%, assay sensitivity of 93%, specificity of 92%, and a 95% confidence interval, based on Buderer’s formula and calculated using the Excel tool developed by Dr. Wan Nor Arifin (Universiti Sains Malaysia) [14]. A minimum of 1,123 specimens was required. From these, 400 samples were randomly selected using a computerized randomization tool (CalculatorSoup Random Number Generator) for multiplex PCR testing. Sampling was evenly distributed across all four seasons to account for seasonal variation in infection patterns [15].

Molecular testing procedures

DNA extraction was performed on 400 whole blood samples using the QIAamp DNA Blood Midi/Maxi Kit (Qiagen, Germany), following the manufacturer’s protocol. Multiplex real-time PCR was carried out using the Fast Track Diagnostic Kit (Luxembourg) on the Sansure SLAN 96 Real-Time PCR System.

Due to limited availability of culture media, the recommended pre-culture enrichment step in Tryptic Soy Broth with 10% Oxgall for Salmonella was omitted. Diagnostic accuracy was evaluated against reference standards, namely blood culture using the BactAlert system for *Salmonella* Typhi/Paratyphi, immunochromatography (BinaxNOW) and peripheral smear microscopy for *Plasmodium* spp., and immunochromatography (Panbio Dengue test) for dengue virus. Sensitivity, specificity, positive predictive value (PPV), and negative predictive value (NPV) were computed for the PCR assay.

Statistical analysis

Data were analyzed using STATA version 17.0 (StataCorp, College Station, Texas). Categorical variables were summarized as frequencies and percentages, and continuous variables as medians with interquartile ranges (IQRs). Multivariate logistic regression was used to identify potential risk factors for infection positivity. Diagnostic performance metrics, including sensitivity, specificity, PPV, and NPV, were manually calculated using standard formulas based on the binomial distribution.

## Results

**Figure 1:**
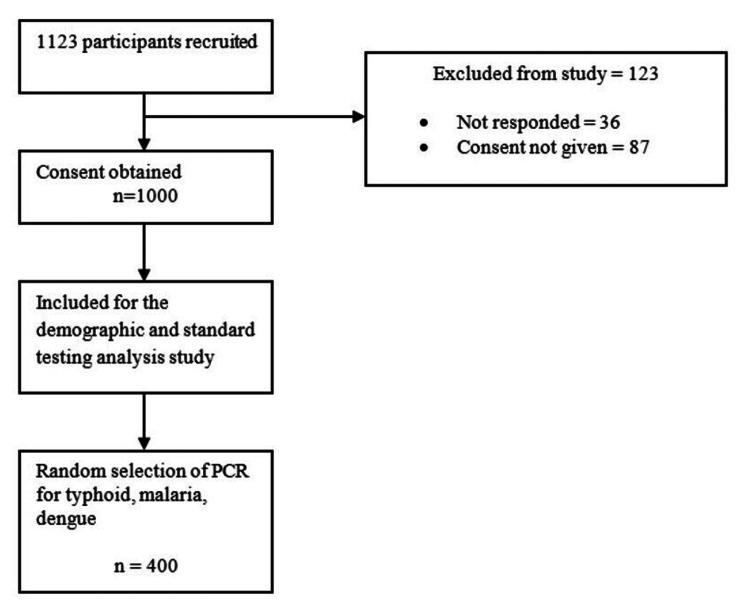
Patient recruitment and selection method for polymerase chain reaction (PCR) testing

During the one-year study period (November 29, 2021, to November 15, 2022), a total of 1123 patient blood samples were collected, with case clusters predominantly from Karachi (Figure 1). 

**Figure 2:**
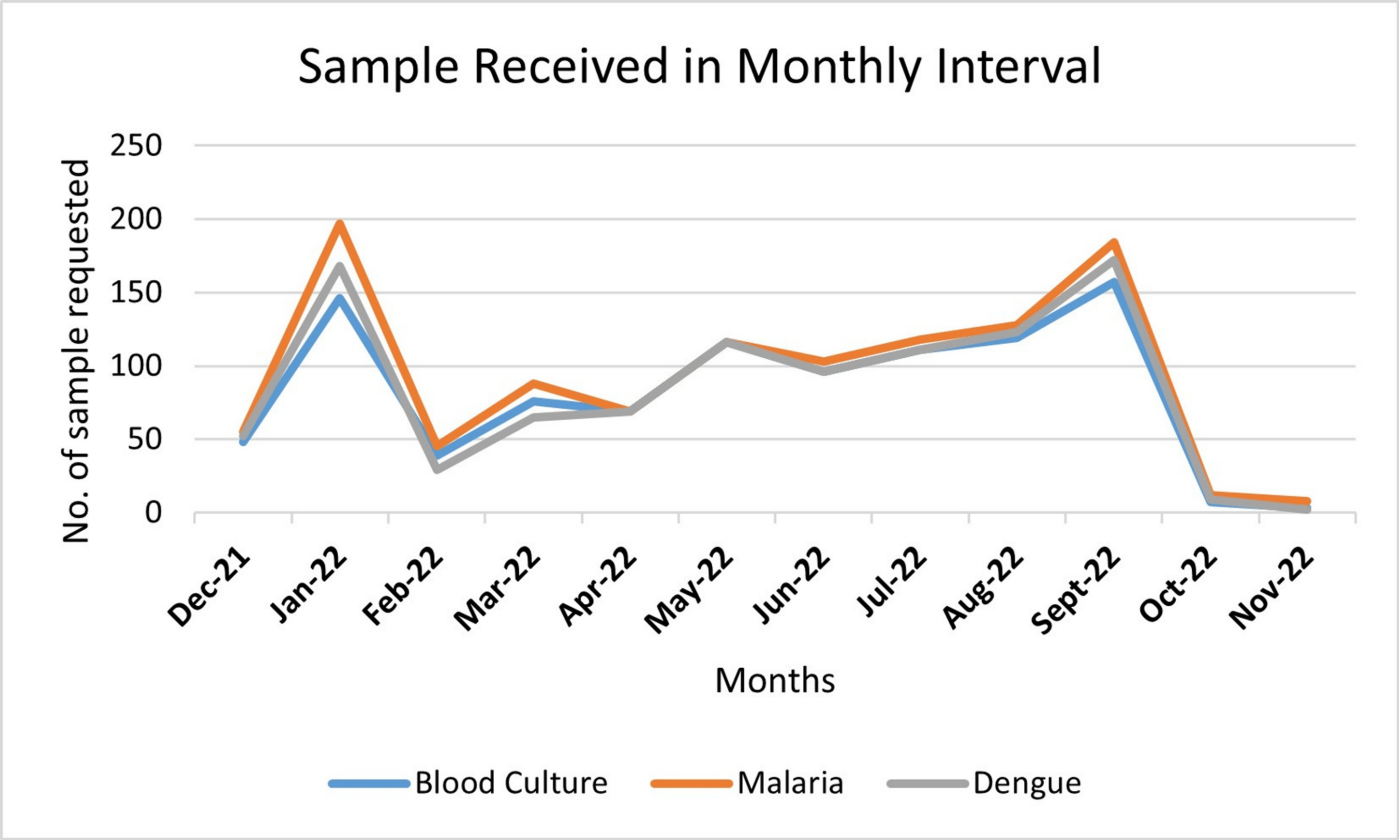
Peak seasons identified for total number of samples received in monthly interval

**Figure 3:**
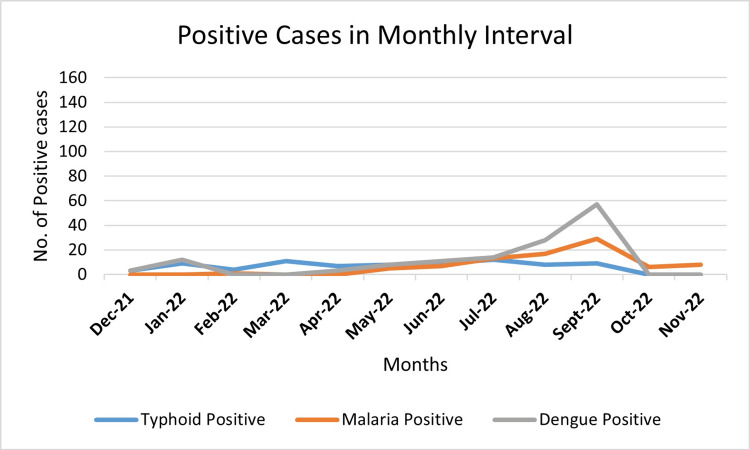
Peak seasons identified for number of samples that turned out positive on standard testing methods

Infections peaked seasonally during monsoon months (July to September), with geographic clustering in Karachi (Figures 2, 3).

Sociodemographic and clinical characteristics of the 1,000 participants are summarized in Table 1. The cohort included 561 males (56.1%) and 439 females (43.9%), with a mean age of 23.00 ± 20.84 years (median 17.00, IQR 5.00-34.00). Fever was present in 87.5% of cases, with a mean temperature of 102.21 ± 0.80 °F and an average duration of 3.05 ± 0.66 days. Most patients (99.3%) sought care at clinics before testing, and 50.8% used antibiotics prior to blood testing (Table 1).

**Table 1:** Demographic and clinical characteristics of study participants Data presented as n (%) for categorical variables and mean ± SD and median (IQR) for continuous variables. SD: standard deviation; IQR: interquartile range.

Characteristics	Frequency (%), N = 1000
Sex	
Male	561 (56.1)
Female	439 (43.9)
Age category	
Infants (0-1 years)	76 (7.6)
Child (2-10 years)	326 (32.6)
Adolescents (11-19 years)	132 (13.2)
Young adults (20-35 years)	235 (23.5)
Adult (36-65 years)	177 (17.7)
Old age adult (above 65 years)	54 (5.4)
Age in years	
Mean ± SD	23.00 ± 20.84
Median (IQR)	17.00 (5.00-34.00)
Symptom category	
Fever	875 (87.5)
Fever and other symptoms	125 (12.5)
Temperature (°F)	
Mean ± SD	102.21 ± 0.80
Median (IQR)	102.00 (102.00-102.00)
Duration of fever in days	
Mean ± SD	3.05 ± 0.66
Median (IQR)	3.00 (3.00-3.00)
Medical care before the test	
Clinic	993 (99.3)
Self-medication	7 (0.7)
Antibiotic use before the test	
Used	508 (50.8)
Did not use	492 (49.2)
Test requested by the physician	
Malaria	34 (3.4)
Blood culture and malaria	73 (7.3)
Malaria and dengue	95 (9.5)
Blood culture, malaria, and dengue	798 (79.8)
Number of tests requested by the physician	
Single	34 (3.4)
Multiple	966 (96.6)
Malaria test status (N = 1000)	
Negative	918 (91.8)
Positive	82 (8.2)
Dengue test status (N = 893)	
Negative	750 (84.0)
Positive	143 (16.0)
Typhoid test status (N = 861)	
Negative	787 (91.4)
Positive	74 (8.6)
Antibiotic use before the test	
Used	183 (18.3)
Used but not known	325 (32.5)
Don't use	492 (49.2)
Antibiotic	
Amoxicillin	9 (4.9)
Azithromycin	117 (63.9)
Azithromycin and imipenem	12 (6.6)
Cefixime	38 (20.8)
Ceftrixone	5 (2.7)
Ciprofloxacin	2 (1.1)
Route of antibiotic	
IV + oral	5 (1.0)
Oral	503 (99.0)
Hospitalization	
Yes	2 (0.2)
No	998 (99.8)
Type of toilet use	
Commode	774 (77.4)
WC	226 (22.6)
Routine insecticides	
No	749 (74.9)
Yes	251 (25.1)

Statistical analyses showed significant associations between infection status and demographic or clinical variables. Age categories were significantly associated with all infections: children aged 2-10 years had higher odds of typhoid (OR 9.9; 95% CI 2.34-41.85; p = 0.002), and adolescents aged 11-19 years had increased odds of malaria (OR 2.53; 95% CI 1.21-5.30; p = 0.013) and dengue (OR 2.72; 95% CI 1.17-6.33; p = 0.02). A higher body temperature was also a significant predictor of malaria (OR 1.57 per °F increase), while self-medication strongly increased risk (OR 16.84; 95% CI 3.24-87.36; p < 0.001), and antibiotic nonuse decreased malaria risk (OR 0.44; 95% CI 0.27-0.74; p = 0.002) (Table 2).

**Table 2:** Multivariate logistic regression analysis P < 0.05 was considered statistically significant. ref. = reference category.

Multivariate logistic regression analysis	Malaria Positive (N = 924)		Dengue Positive (N = 893)		Typhoid Positive (N = 816)	
	OR (95 % CI)	P-Value	OR (95 % CI)	P-Value	OR (95 % CI)	P-Value
Age category						
Infants (0-1 years)	NA		Ref.		7.40 (1.47, 37.13)	0.015
Child (2-10 years)	Ref.		1.10 (0.50, 2.57)	0.752	9.91 (2.34, 41.85)	0.002
Adolescents (11-19 years)	2.53 (1.21, 5.30)	0.013	2.72 (1.17, 6.33)	0.02	6.86 (1.47, 31.96)	0.014
Young adults (20-35 years)	1.98 (1.01, 3.90)	0.046	1.48 (0.65, 3.37)	0.345	3.97 (0.86, 18.17)	0.076
Adult (36-65 years)	2.69 (1.34, 5.39)	0.005	1.26 (0.53, 2.99)	0.594	Ref.	
Old age adult (above 65 years)	2.09 (0.75, 5.84)	0.158	0.70 (0.19, 2.49)	0.587	NA	
Temperature (°F)	1.57 (1.20, 2.05)	0.001	0.72 (0.56, 0.93)	0.013		
Medical care before the test						
Clinic	Ref.					
Self-medication	16.84 (3.24, 87.36)	<0.001				
Antibiotic use before the test						
Used	Ref.				Ref.	
Did not use	0.44 (0.27, 0.74)	0.002			0.22 (0.12, 0.40)	<0.001

Among the 400 PCR-tested samples, 211 (52.8%; 95% CI 47.8-57.7) were positive for at least one infection. Among the routine blood tests requested for all three infections (*Salmonella*, malaria, and dengue), 312 samples included 60 *Salmonella* spp.-positive, 8 malaria-positive, and 70 dengue-positive cases by reference tests. PCR testing identified 24 *Salmonella* spp., 30 malaria, 36 dengue, and 1 case positive for all three pathogens within this group, which was missed by the reference test.

In 48 samples tested for malaria and dengue, 33 malaria and 6 dengue cases were detected by the reference methods. PCR detected 30 malaria and 3 dengue cases in this group. Among the eight samples tested for *Salmonella *spp. and malaria, both the reference tests and PCR showed the same results. Lastly, in three samples tested for *Salmonella *and dengue, no pathogens were identified by reference testing alone, but PCR detected one *Salmonella *spp. Coinfections were identified in six patients, including the malaria-dengue group by the reference method, while one case of typhoid-dengue-malaria was detected by the PCR method. PCR identified 16 additional malaria cases that were missed by reference methods (Table 3).

**Table 3:** Relevance of test results with PCR outcomes Data are presented as frequencies. The table compares conventional reference test results (culture for *Salmonella*, microscopy/ICT for malaria, NS1 antigen and IgM for dengue) with molecular outcome confirmed by PCR. PCR: polymerase chain reaction, ICT: immunochromatographic test, NS1: structural protein 1 antigen test, IgM: immunoglobulin M.

Multi-test Request	Reference Test Results				PCR Results			
	No. of Coinfection	*Salmonella* (Culture)	Malaria (ICT, microscopy)	Dengue (NS-1, IgM)	No. of Coinfection	Salmonella	Malaria	Dengue
*Salmonella *spp.+ malaria + dengue (n = 312)	0	60	8	70	1	24	30	36
Malaria + dengue (n = 48)	6	0	33	6	0	0	30	3
*Salmonella *spp. + malaria (n = 8)	0	1	10	0	0	1	10	0
*Salmonella *spp. + dengue (n = 3)	0	0	0	0	0	1	0	0

The diagnostic accuracy of PCR in comparison with the reference standards is presented in Table 4. Sensitivity, specificity, PPV, and NPV for PCR were calculated manually using standard formulas based on the binomial distribution. For malaria, PCR demonstrated high diagnostic performance with a sensitivity of 92.4% (95% CI 84.1-97.2%) and a specificity of 98.4% (95% CI 96.8-99.3%). The PPV was 90.7% (95% CI 81.7-95.9%), while the NPV was 98.6% (95% CI 97.1-99.4%). For dengue, PCR yielded a sensitivity of 35.3% (95% CI 25.4-46.6%) and a specificity of 94.6% (95% CI 92.2-96.4%), with corresponding PPV and NPV of 78.3% (95% CI 62.3-88.6%) and 71.2% (95% CI 67.0-75.1%), respectively. For typhoid, the sensitivity was 25.0% (95% CI 16.1-36.6%) and the specificity was 93.0% (95% CI 90.2-95.1%), with a PPV of 46.2% (95% CI 31.5-61.7%) and an NPV of 83.7% (95% CI 80.4-86.6%).

**Table 4:** Positive predictive value (PPV), negative predictive value (NPV), sensitivity and specificity for malaria, dengue and typhoid Values are reported as percentages (%). These diagnosis accuracy metrics were calculated by comparing reference test results against polymerase chain reaction (PCR)-confirmed outcomes for each infection.

S.no	Infection	PPV	NPV	Sensitivity	Specificity
		%	%	%	%
1	Malaria	90.7	98.6	92.40	98.41
2	Dengue	78.26	71.2	35.3	94.6
3	Typhoid	46.23	83.7	25	93

## Discussion

This study revealed the highest sensitivity (92.40%), specificity (98.41%), and positive and negative predictive values (90.7% and 98.6%, respectively) for detecting malaria. The multiplex PCR kit’s specificity for dengue and typhoid was also high (94.57% and 93%, respectively). However, the sensitivity for both dengue and typhoid was very low (35.29% and 25%, respectively). The possible reason for the lowest sensitivity for typhoid was our deviation from the kit protocol in cases of suspected typhoid due to the unavailability of media, which recommended the use of precultured whole blood in tryptic soy broth (TSB) with 10% Oxgall medium for five hours at 37°C before DNA extraction. The low sensitivity observed for dengue in our multiplex PCR may be attributed to factors such as low viral loads in sampled febrile patients and the timing of sample collection relative to infection onset.

The novelty of our study is that it is among the first validations of multiplex PCR for the diagnosis of febrile illnesses, including malaria, dengue, and typhoid, in Pakistan. This work fills a significant diagnostic gap in the region, where limited molecular diagnostic data currently exist. By providing evidence of the assay's diagnostic accuracy in a setting with a high burden of tropical infections, our study contributes novel insights with potential applicability both regionally and in other tropical countries facing similar diagnostic challenges. More importantly, the multiplex PCR assay successfully identified samples that tested negative by the reference method for all three infections, emphasizing the critical role of multiplex PCR in diagnostic interpretation and highlighting its efficiency in managing clinical uncertainties.

The study identified a relationship between age, sex, and symptoms of infections. For example, malaria occurred significantly in adolescents and adults and was associated with fever, abdominal pain, vomiting, and headache. Typhoid fever was most prevalent among infants, children, and adolescents, with the age group 2 to 10 years accounting for the highest percentage of cases (56.8%). The higher prevalence of typhoid in children, especially those under five, may be related to the maturation of the immune system, as younger children exhibit quantitatively lower or less sustained humoral and cellular immune responses to *Salmonella* Typhi compared to older children and adults. However, this remains an area for further investigation and should be considered a hypothesis supported by current immunological evidence rather than a definitive conclusion.

Self-medication was common for all three infections (malaria, dengue, and typhoid), while a significant association was observed with malaria and dengue. Azithromycin was observed as a frequently used antibiotic to treat suspected typhoid cases. Most cases were managed on an outpatient basis, with only two requiring hospitalization, which indicates that mild to moderate cases were successfully treated without the need for hospital admission.

Fever was the most prominent symptom of acute illness, with temperature typically peaking for approximately two days in most patients. Some patients also experienced abdominal pain, headache, vomiting, or diarrhea along with fever. Most tests requested were two or more, as shown in Tables 1, 3. These findings are consistent with those of previous studies in Pakistan, which identified malaria, dengue, and typhoid as the primary causes of acute febrile illness and reported cases as separate infections or coinfections, such as malaria with typhoid or malaria with dengue [16-17].

We noted a striking demand for malaria testing, with 1,000 prescriptions during the study period. This statistic points toward a heightened clinical suspicion of malaria among healthcare practitioners and the considerable prevalence of malaria within our region. This finding is supported by the alarmingly high number of malaria cases reported in Pakistan by the WHO in 2022. According to this report, 2.6 million malaria cases occurred in 2021, which increased to 3.4 million suspected cases in 2022 (January to August). Interestingly, 78% of these cases occurred in the provinces of Sindh and Baluchistan (WHO 2022) [18].

A similar finding was also identified for dengue patients. Our study revealed an increase in dengue cases in August and September, indicating a remarkable tendency for disease outbreaks during the post-monsoon period, when environmental conditions may contribute to increased transmission (Figure 2). This could again be related to the catastrophic flooding in June 2022, resulting in an increase in dengue cases compared with previous years, with a case fatality rate (CFR) of 0.25% (WHO 2022) [9].

Furthermore, the XDR typhoid surge in Pakistan has raised concerns about causative agents of public health crises, morbidity, and mortality. The XDR *Salmonella* Typhi strains circulating in Pakistan are resistant to ampicillin, chloramphenicol, trimethoprim-sulfamethoxazole, ciprofloxacin, and ceftriaxone. These resistances severely limit treatment options, leaving azithromycin and carbapenems (such as meropenem and imipenem) as some of the few remaining effective drugs [19-21]. All these concerns emphasize the need for a single effective test that can detect agents of febrile illness simultaneously. Given the complexity and time-consuming nature of multiple diagnostic tests, this approach can contribute to an increased workload and diagnostic challenges for medical professionals. Therefore, there is an urgent need to develop and integrate rapid, reliable, and single tests capable of simultaneously detecting multiple pathogens involved in febrile illnesses.

The multiplex PCR assay demonstrated notable advantages in terms of turnaround time and cost-effectiveness. The average time to result was approximately 4 to 5 hours, significantly faster than conventional methods such as blood culture (which can take 24 to 48 hours). Although the upfront cost of PCR reagents is higher, the per-test cost becomes comparable or even lower when multiple pathogens are screened simultaneously within a single reaction, reducing the need for repeated individual tests and technician time. The demonstrated diagnostic performance supports the integration of multiplex PCR into national surveillance and hospital laboratory networks, where it could enhance outbreak detection and guide timely, evidence-based treatment of febrile illnesses.

Strengths of the study

To our knowledge, this study is among the first in Pakistan to validate a commercial multiplex PCR assay for the simultaneous detection of *Salmonella *Typhi/Paratyphi, *Plasmodium *spp., and dengue virus in patients with acute febrile illness. It addresses a major diagnostic gap in a region where conventional methods are time-consuming and limited in sensitivity. The study followed a prospective cross-sectional approach over a complete one-year period, allowing for an accurate assessment of seasonal variation, disease clustering, and infection trends across monsoon and non-monsoon months. Our study provides a robust dataset covering both pediatric and adult populations, strengthening the generalizability of findings. Lastly, samples were collected from routine diagnostic workflows at a major tertiary care center, reflecting true clinical practice rather than ideal laboratory conditions.

Limitations of the study

This study has several limitations. First, we included participants only from Sindh and Karachi; therefore, the study findings may not represent Pakistan as a whole. More studies involving vast geographical areas with advanced molecular diagnostic panels should be conducted to obtain true data, demographics, and epidemiology of these tropical infections. As we deviated from the protocol, we observed low sensitivity for typhoid. For the most accurate determination, the sensitivity and specificity of typhoid precultured in Oxgall medium should be assessed. Finally, the current research was aimed primarily at optimizing diagnostic capabilities; as such, we did not pursue tracking the outcomes of patients who tested positive for infections. This area remains a potential focus for future studies to better understand the implications of our diagnostic advancements in patient care.

## Conclusions

This study validates the substantial burden of acute tropical infections, particularly malaria, dengue, and typhoid, in Pakistan. It underscores the urgent need for rapid, accurate diagnostic tools that can identify these infections simultaneously. The commercial multiplex PCR kit demonstrated strong performance for malaria detection, with 92.4% sensitivity and 98.4% specificity, but showed markedly lower sensitivity for dengue (35.3%) and typhoid (25%), despite high specificity. These findings highlight key methodological constraints, including challenges with protocol adherence for typhoid and the assay’s limited sensitivity for dengue, which must be addressed to improve diagnostic accuracy. Early and appropriate investigations can significantly reduce misdiagnosis and prevent unnecessary antibiotic use, supporting antimicrobial stewardship.

To enhance public health impact, integrating such multiplex molecular diagnostics into national surveillance systems, laboratory networks, and community-level outbreak response programs is critical. This approach can facilitate timely, targeted patient management and monitoring of infection trends. Future research should focus on refining assay sensitivity and specificity through the development of protocol-adherent PCR or next-generation multiplex platforms. Additionally, evaluating the cost-effectiveness, scalability, and operational feasibility of these diagnostics in resource-limited settings will be essential to inform widespread implementation and improve clinical and public health outcomes.

## References

[1] Ali N, Khan NU, Waheed S, Mustahsan S (2020). Etiology of acute undifferentiated fever in patients presenting to the emergency department of a tertiary care center in Karachi, Pakistan. Pak J Med Sci.

[2] Chan YC, Salahuddin NI, Khan J. (1995). Dengue haemorrhagic fever outbreak in Karachi, Pakistan, 1994. Trans R Soc Trop Med Hyg.

[3] Hussain A, Satti L, Hanif F, Zehra NM, Nadeem S, Bangash TM, Peter A (2019). Typhoidal Salmonella strains in Pakistan: an impending threat of extensively drug-resistant Salmonella Typhi. Eur J Clin Microbiol Infect Dis.

[4] Bala A, Singh K, Chhabra A, Sidhu SK, Oberoi L (2025). Incidence of dengue, chikungunya, malaria, typhoid fever, scrub typhus, and leptospirosis in patients presenting with acute febrile illness at a tertiary care hospital, Amritsar. J Vector Borne Dis.

[5] Bilal W, Qamar K, Abbas S, Siddiqui A, Essar MY (2022). Infectious diseases surveillance in Pakistan: challenges, efforts, and recommendations. Ann Med Surg (Lond).

[6] (2024). World Malaria Report 2015. https://iris.who.int/bitstream/handle/10665/200018/9789241565158_eng.pdf.

[7] (2025). Common Management Unit for Malaria Control (CMU). Malaria Epidemiological Report 2023. Malaria Epidemiological Report.

[8] Taqi N (2025). Coalition Against Typhoid Surveillance Report 2023. https://www.coalitionagainsttyphoid.org/wp-content/uploads/2024/01/2-Typhoid-presentation_Nada-Taqi_7-Dec-2023.pdf.

[9] (2024). World Health Organization. Dengue - Pakistan. Disease outbreak news. https://www.who.int/emergencies/disease-outbreak-news/item/2022-DON413.

[10] Alied M, Salam A, Sediqi SM, Kwaah PA, Tran L, Huy NT (2023). Disaster after disaster: the outbreak of infectious diseases in Pakistan in the wake of 2022 floods. Ann Med Surg (Lond).

[11] Sea-Liang N, Sereemaspun A, Patarakul K (2019). Development of multiplex PCR for neglected infectious diseases. PLoS Negl Trop Dis.

[12] Frickmann H, Wegner C, Ruben S (2019). Evaluation of the multiplex real-time PCR assays RealStar malaria S&amp;T PCR kit 1.0 and FTD malaria differentiation for the differentiation of Plasmodium species in clinical samples. Travel Med Infect Dis.

[13] Ramanan P, Bryson AL, Binnicker MJ, Pritt BS, Patel R (2018). Syndromic panel-based testing in clinical microbiology. Clin Microbiol Rev.

[14] Buderer NM (1996). Statistical methodology: I. Incorporating the prevalence of disease into the sample size calculation for sensitivity and specificity. Acad Emerg Med.

[15] Furey Furey, Edward "Random (2022). Random Number Generator from Calculator Soup. https://www.calculatorsoup.com/calculators/statistics/random-number-generator.php.

[16] Khan MA, Mekan SF, Abbas Z, Smego RA Jr (2005). Concurrent malaria and enteric fever in Pakistan. Singapore Med J.

[17] Qureshi AW, Khan ZU, Khan L, Mansoor A, Minhas R (2019). Prevalence of malaria, typhoid and co-infection in district Dir (Lower), Pakistan. Biosci J.

[18] (2024). World Health Organization. Malaria - Pakistan: Disease Outbreak News. https://www.who.int/emergencies/disease-outbreak-news/item/2022-DON413.

[19] Fatima M, Kumar S, Hussain M (2021). Morbidity and mortality associated with typhoid fever among hospitalized patients in Hyderabad District, Pakistan, 2017-2018: Retrospective record review. JMIR Public Health Surveill.

[20] da Silva KE, Tanmoy AM, Pragasam AK (2022). The international and intercontinental spread and expansion of antimicrobial-resistant Salmonella Typhi: a genomic epidemiology study. Lancet Microbe.

[21] Irfan S, Hasan Z, Qamar F (2023). Ceftriaxone resistant Salmonella enterica serovar Paratyphi A identified in a case of enteric fever: first case report from Pakistan. BMC Infect Dis.

